# Patient perception of consent processes for epidural analgesia in induction of labour: a qualitative study

**DOI:** 10.1111/anae.16637

**Published:** 2025-05-12

**Authors:** Danna Nitzani, Jacqueline Nicholls, Katherine Maslowski, Robert Craig, Sohail Bampoe, Melissa Whitten, Anne Lanceley

**Affiliations:** ^1^ Department of Reproductive Health UCL EGA Institute for Women's Health, University College London London UK; ^2^ Department of Anaesthesia and Perioperative Medicine University College London Hospitals NHS Foundation Trust London UK; ^3^ Centre for Perioperative Medicine University College London London UK; ^4^ Cleveland Clinic Lerner College of Medicine, Case Western Reserve University Cleveland OH USA; ^5^ Women's Health Division University College London Hospitals NHS Foundation Trust London UK; ^6^ Department of Maternal and Fetal Medicine EGA Institute for Women's Health, University College London London UK; ^7^ Department of Women's Cancer EGA Institute for Women's Health, University College London London UK

**Keywords:** consent, epidural analgesia, induction of labour

## Abstract

**Introduction:**

Women undergoing induction of labour often utilise epidural analgesia. Obtaining consent for labour epidural presents a unique challenge for the obstetric anaesthetist, who must comply with the legal standards of consent. This study explores how women perceive the consent process for epidural analgesia during induction of labour.

**Methods:**

This was a qualitative, single‐centre, interview‐based study. Fourteen women who received an epidural for labour analgesia were interviewed using a semi‐structured interview guide. Data were analysed using thematic analysis.

**Results:**

Four themes described women's experience of the consent process. Understanding alternatives, risks and benefits; for example, time constraints hindering the effective communication of information around epidural analgesia, including alternative analgesic options. Timing of information; for example, the value of information was diminished by pain, fatigue and the imminence of the procedure. Timing of consent; for example, physiological and psychological demands of labour negatively impacted patients' ability to engage with the consent process. Anaesthetists' assessment of patient understanding; for example, confirmation of patient understanding by anaesthetists was lacking.

**Discussion:**

Women's experiences of the consent process for induction of labour suggest that in the context of the pain and exhaustion of labour, inadequate and untimely information provision and dialogue between women and their anaesthetists can undermine the implementation of lawful consent.

## Introduction

Induction of labour is performed in approximately one‐third of births in the UK [1]. It is associated with increased epidural use when compared with spontaneous labour; a survey of women in the UK reported that 47% of women undergoing labour induction had an epidural [2]. Guidance from the National Institute for Health and Care Excellence recommends that women undergoing induction of labour should be offered an epidural before starting oxytocin, or later if requested [3].

Lawful patient consent to treatment is an immutable requirement of all healthcare practice. The right of every woman to share in decision‐making is key to patient‐centred maternity care. These rights were affirmed in the landmark ruling of *Montgomery vs. Lanarkshire*. The case concerned a woman who was ill informed about the risks of vaginal birth. The significance of the case lies in its ruling that patients must be informed of any ‘material’ risks where what is ‘material’ is defined subjectively by reference to what matters to the particular patient or objectively, what a reasonable person in their position would likely deem significant [4]. To incorporate the wishes of an individual patient into the consent process effectively, Montgomery and subsequent cases [5, 6] emphasise the centrality of genuine dialogue with the patient in which the patient's central role in decision‐making is supported actively. Fostering such dialogues, which are autonomy supporting, requires commitment and skill to understand what matters to an individual woman and to enable a woman's participation irrespective of her circumstances.

Limited evidence on consent to epidural in maternity care indicates deficiencies in information provision [7], poor understanding of risks [8] and, in one study, a concerning absence of any form of consent [9]. Anecdotally, there is a degree of professional unease among some anaesthetists about the consent process for epidural in the context of induction of labour, where severe pain may influence a woman's decision‐making ability. Even less is known about consent in this situation, although limited published work indicates that consent is at best brief and uninformative [10] and hampered by pain [10, 11, 12, 13]. This study aimed to explore how women experience consent to epidural pain relief for induction of labour.

## Methods

This qualitative study used semi‐structured interviews to explore patient experiences of consent to epidural in induction of labour. Ethical approval was granted by the UK Health Research Authority and the study is reported with reference to COREQ [14].

The study took place in a large inner city NHS Foundation Trust hospital. Women were eligible to take part if they were: primiparous; aged ≥ 18 y; had consented to epidural analgesia before or during induction of labour; and were able to understand written and spoken English. Individuals were not included if neuraxial block was performed for surgical intervention rather than labour analgesia; they lacked mental capacity to participate; or were too unwell to participate. Multiparous women were not included to limit the impact of previous experience(s).

Consecutive data sampling was used, whereby women who underwent any form of induction of labour and consented to epidural were identified and approached by midwives on the labour or postnatal ward. If they were willing to hear about the study, they were introduced to a member of the research team (DN), who provided verbal and written information. All participants provided written consent before being interviewed. Demographic data were collected, including self‐reported ethnicity.

All interviews were conducted by DN and audio recorded face‐to‐face in private rooms to ensure confidentiality. Before interviewing, DN completed training for conducting semi‐structured interviews led by the research team (AL and JN) who are experienced qualitative researchers. Recordings were anonymised and transcribed verbatim by a confidential transcription service.

Based on similar studies in maternity care [15, 16], we anticipated we would need to recruit 12–14 women. An interview schedule (online Supporting Information Appendix S1) was developed by the research team previously (AL and JN) and adapted for this study by DN, AL and JN with input from a consultant anaesthetist (SB) to include specific questions concerning the information women were provided with regarding epidural analgesia. To minimise social desirability bias, the interview schedule was developed to encourage participants to share excerpts of their conversations about consent, rather than binary answers.

Inductive thematic analysis was conducted following Braun and Clarke's six‐step framework [17]. Our analytic interpretation was underpinned by legal theory which concerns the practical problems of law, approached from different disciplinary perspectives [18]. After initial reading and re‐reading, line‐by‐line manual coding of all the transcripts was conducted by DN and reviewed by the research team. Themes were refined until a final set was agreed. Data saturation was reached when no new insights emerged from our analysis. We also considered saturation respecting the theoretical development and adequacy of our results and whether there was sufficient expansion of each thematic category to determine its characteristics and nuanced meanings. Data collection continued until we had analysed 14 interviews and, with those, we judged we had achieved conceptual depth and there were no new emerging themes identified. For quality assurance, the research team met regularly to discuss extracts of coded transcripts and arising themes and any discordance was resolved.

## Results

Twenty‐four women were approached face‐to‐face to take part and 14 women were interviewed between January and April 2023. Ten declined to participate primarily due to pain, fatigue or a concern about the interview length. Interviews occurred from 14 to 83 h after delivery, with one exception of 131 h due to the patient developing sepsis. Interviews lasted between 14 and 35 min, with an average length of 24 min. Participant characteristics are presented in Table 1.

**Table 1:** Characteristics of patients (n = 14) receiving epidural analgesia before or during induction of labour. Values are number.

**Age; y**	
25–29	3
30–34	6
35–39	5
**Ethnicity**	
White, British	4
White, other	6
British, Bangladeshi	1
Asian, Bangladeshi	1
Other Asian background	1
Other Black background	1
**Level of education**	
Secondary school	1
Higher education qualification below degree	1
Degree	8
Higher degree	4
**Method(s) of induction**	
ARM and syntocinon	4
Dinoprostone and ARM	1
Dinoprostone, ARM and syntocinon	5
Dinoprostone and syntocinon	2
Syntocinon	2
**Indication(s) for induction** ^†^	
Gestational diabetes mellitus	4
Pre‐eclampsia toxaemia	2
Prelabour rupture of membranes	2
Prolonged latent phase	3
Reduced fetal movements	5
Small for gestational age	3
**Mode of delivery**	
Caesarean section	7
Instrumental	4
Non‐assisted vaginal	3

ARM, artificial rupture of membranes.

^†^ More than one indication possible.

Thematic analysis identified four overlapping themes: understanding alternatives, risks and benefits; timing of information; timing of consent; and assessment of patient understanding by anaesthetists. Direct quotes from participants are presented below to illustrate the themes, where each participant is assigned a number.

Participants reported limited discussion about alternatives to epidural analgesia. The instigation of epidural‐centred discussions varied between women. Most participants accepted or requested an epidural based on their previous knowledge and perceived that they had limited opportunities to discuss alternatives:

*“More than saying (…) ‘here are the six (pain relieving strategies), which one you want’, they were like, ‘you've read about it already, what are you thinking?’”* Patient 7

*“I brought it [epidural] up so again it's different because it wasn't like ‘here are your options, what would you like?’ (…) I would have listened, yes*.*”* Patient 5

Participants perceived that once the decision for induction of labour had been taken, there was a protocol which dominated the decision‐making process to induction of labour. This restricted the time available for consideration of alternative pain relief, with some alluding to an overemphasis on epidural during induction of labour as compared with alternatives.

*“There was a lot of procedure in place, there wasn't a, like a time to kind of like think about other alternatives (to epidural), so ‘this is the induction process, this is what you're getting, these are the time frames’ and stuff like that.”* Patient 13

*“The focus had always been on the epidural at the point that we were in active labour (…) One thing we still don't know actually, is whether – what the other pain relief options were. If we'd wanted to go down the opioid route, could you do that if you been induced?”* Patient 11

Participant responses also revealed inadequate scope and discussion of information regarding the risks and benefits of epidural. Although most participants reported being informed about the risks, their responses when asked about specific risks, such as headaches and nerve damage, occasionally revealed the contrary. Participants also perceived that beyond being informed of the facts of the risks, there was little opportunity for them to voice their uncertainty and fears about them. If they did express worries, they felt these were downplayed by anaesthetists:

*“So, when I read some papers, even though the risk is very rare, I felt some afraid. (…) But doctor (anaesthetist) advised me that's really rare or that's never happened, but in the textbook, so they have to explain.”* Patient 10

*“The risk factors were quite scary, while you're already like uncomfortable it's like you're signing yourself into like a long‐term future, potential damage.”* Patient 13

Most participants perceived a lack of discussion around the risks and benefits of not having an epidural and could not recall an example of a risk when they were asked to provide one. Of those, some believed that a discussion on this topic was unnecessary:

*“I can't remember if it was discussed or not but I don't think, if it wasn't discussed it wasn't needed.”* Patient 6

*“It was more focused on the risks of having one rather than the risks of not having one or the risks of – or the benefits of – not having one”*. Patient 11

Most women initiated a request for an epidural, and only a few patients were offered one. Of those requesting an epidural, most asked for it in anticipation of, rather than due to, substantial pain. Thus, it is unlikely that, for the majority of participants, substantial pain hindered their ability to engage in meaningful discussion at the stage of requesting an epidural. Pain contributed as a larger distractor at the point of formal consent, which differed from the point when the epidural was initially discussed.

Most participants were provided with a leaflet containing statistics on the different risks associated with epidural analgesia immediately before formally consenting. By this stage, they felt that the value of the written information was diminished by pain even when the leaflet was read aloud by a birth partner. Several participants reported telling their anaesthetist that they had read the leaflet when they had not, to prevent delays in receiving the epidural:

*“He (anaesthetist) just said ‘read the card’ and obviously when you're in pain you're not really reading things in much detail, you're just skimming through. I'm like, ‘Yeah I read it.’ (…) you don't actually read the card.”* Patient 1

Among the minority of women who received written information shortly after their admission to the labour ward, some recognised that the opportunity to think about the information facilitated their informed decision making:

*“But they weren't strong contractions because I still needed the hormone drip because of going through the induction process. So I was still able to read it and make an informed decision about it.”* Patient 11

*“It [epidural] wasn't on the table but she [midwife] gave me that [leaflet] to read in advance (…) I think it was really good that they gave me that document before the whole thing had started because once it starts you just don't know where your head is at.”* Patient 8

Participants described being distracted by physiological and psychological demands associated with labour whilst formally consenting to epidural. Pain, opioids and extreme fatigue were the principal diverting factors:

*“Later on I was offered [epidural] again and I accepted, yes. That was a blur because remember I had the pethidine and I was absolutely drugged up and off of my face.”* Patient 6

*“I think the time at which they gave me the epidural probably was like the worst possible time that the anaesthetist could have for themselves. (…) I was like semi‐conscious really. [laugh]”* Patient 3

Most participants stated they had opportunities to ask the anaesthetist questions, whilst some participants reported that discussions were not facilitated at the point of formally consenting. However, several doubted the effectiveness of these discussions due to their timing:

*“The anaesthetist is called, they bring the sheet of paper, you sign it and then it's in. There isn't really that middle ground to discuss.”* Patient 11

*“At the point of which I was consenting to the epidural I'm not sure I was really in the right head space to like ask a lot of question.”* Patient 3

Some participants refrained from asking questions as they perceived it would delay epidural administration:

*“They asked like, ‘Do you have any questions?’ and I was like, ‘No’ because obviously I was just like, ‘just give me what I need’.”* Patient 1

When discussions regarding the risks and benefits of epidural occurred with anaesthetists, the ability of participants to fully engage was often overshadowed by extreme pain such that some women expressed a willingness to consent regardless of any information received at this point:

*“And then right before I had it [epidural] done the anaesthetist also talked through the risks in detail to both me and my partner. In saying that I was in pretty heavy contractions at that point so honestly I would have done anything to make them stop.”* Patient 8

*“They said so many [risks], those were the only two things I cached [caught] up on, because I was like ‘I don't care,’ I would rather not feel pain right now.”* Patient 12

Most participants reported that their understanding of epidurals was not assessed by the anaesthetist before siting the epidural. The few women who perceived it was assessed quoted examples that failed to illustrate this, including referencing safety checks instead:

*“Yes they checked my understanding (…) I think they ask like, ‘What's your name? What's your date of birth?’ and ‘What treatment are you getting now?’ (…) they just like specifically ask these three questions like they know you're aware you're here (…) just like you're conscious or something.”* Patient 2

*“It was more that they just told me and then asked if I was happy with it rather than checking that I understood.”* Patient 3

The research team was mindful of the influence of our researcher positionality on the research process [19]. We were conscious of the potential strengths and biases of our sex, cultural background, profession and the influence of our personal and professional expectations of maternity care delivery as the research progressed. Members of the study team (KM, MW) are female doctors and DN is a female medical student. Female researchers AL and JN trained and practised as healthcare professionals; JN is also a trained solicitor with experience in health law; and SB and RC are male anaesthetists. We negotiated our different and shifting perspectives during team conversations, which were informed by reflexive insights from DN's fieldnotes. Through such questioning engagement, we aimed to avoid recognised bias and ensure fidelity to the data.

## Discussion

Our results suggest that women experience limitations to the provision of timely information and may not be able to retain the information given due to pain or other factors. This aligns with the findings of a previous review, which considered studies which pre‐date the Montgomery judgement and concluded that consent for epidural in labour is often inadequately informed and treated as a mere formality [6]. In the context of the more stringently articulated consent requirements of Montgomery consent and of their endorsement in the intra‐partum context [20], we suggest that our findings are particularly worrisome.

Consent and choice are indisputable tenets of maternity care [21]. Despite this, most women in this study did not perceive they had been fully appraised of the risks, benefits or alternatives to proceeding with an epidural or choosing not to do so [22].

The evolving clinical scenario of induction of labour can impose significant time constraints on the consent process. Chrimes and Marshall went so far as to say that informed consent in anaesthesia is an ‘illusion’ due to the gap between its principles and the realities of time pressured clinical practice [23]. To obviate the practice realities, the Association of Anaesthetists recommends that advice about labour analgesia should be provided in early pregnancy as well as at the time of the procedure [24]. Within the anaesthetic community there is an acknowledgement of anaesthetists' presumptive reliance on information provided earlier in the care pathway [25, 26]. The results of a UK survey of anaesthetists likely evidences this reliance as reflected in the significant variation in the range and likelihood of epidural complications discussed with women before consenting to epidural analgesia in labour [26]. However, enacting genuinely lawful informed consent requires that discussion concerning a woman's preferences and understanding of the risks and benefits of the procedure must be revisited at the time of consent.

Related to the issue of reliance on information offered previously is the extent to which anaesthetists may delegate responsibility for consent to other doctors or healthcare professionals such as midwives. The UK law and professional guidance states unequivocally that any delegating doctor is liable for the lawful exercise of the professional and legal responsibilities of consent [27]. In the context of consent for epidural analgesia, Cole et al. cautions anaesthetists in relation to delegating consent as they evidenced little dialogue or ‘negotiation’ between midwives and women. On the contrary, women in the study had a ‘unilateral’ responsibility for the decision with little input from the midwife [28].

We were concerned that most women in our study reported insufficient dialogue with anaesthetists and midwives on the risks, benefits and alternatives to epidural analgesia. Fostering genuine shared decision‐making dialogue is undoubtedly professionally challenging and it may be that healthcare practitioners are unwilling or lack the requisite skills [29, 30]. Indeed, the findings of an Australian ethnographic study suggests the deficiencies and incompleteness of the consent process for epidural analgesia may be because neither midwives nor anaesthetists claim full “*ownership of the task*” [10].

Our findings also echo those of previous studies which have revealed deficiencies in the communication of the risks and benefits of epidural in labour. Whereas some surveys have shown no, or very poor, recall of important epidural risk‐related information conveyed during labour [7, 8, 9], other studies report excellent recall of risks, particularly when assessed soon after epidural insertion [31, 32]. Interestingly, some studies report that despite excellent recall of information, many women self‐rated their understanding of the risks as poor and expressed a desire for better information provision ahead of labour [32, 33]. We consider that these various findings serve as a reminder that the communication of risk is a subtle and complex professional endeavour that goes beyond mere information provision.

Despite the evolving nature of intrapartum care, we were also concerned that most women perceived a lack of discussion of alternatives to epidural once they were admitted to the labour ward. Asking a patient to make a decision requiring consent when in the throes of labour is recognised to be challenging [34]. However, rather than justifying any failure to ensure a patient wants to proceed with epidural analgesia, it underscores the need to ensure women appreciate they have a genuine choice in order to comply with the legal injunctive to discuss reasonable alternatives [35]. As noted in *Wyatt v Curtis*, it is unreasonable to expect women to instigate conversations on alternatives: “*there is arguably something unreal about placing the onus of asking upon a patient who may not know that there is anything to ask about*” [36].

Almost half of the women in our study recognised that opioid medication, pain or fatigue, hindered their ability to engage with the consent process and this was reflected in an unwillingness to ask questions and a diminished ability to listen to, or read, information. The study by Fröhlich et al. supports this, as 79% of the women they interviewed perceived that discomfort due to labour impacted their ability to provide informed consent [33]. Qualitative evidence suggests that obstetricians and midwives in the labour ward setting have concerns regarding the decision‐making abilities of some women, if they are in physical pain or psychological (di)stress [37]. This ‘grey area’ of capacity to consent, whereby both the women and those providing their care doubt the validity of their consent, requires closer examination.

Overall, our study reveals some worrying aspects of consent in the context of epidurals in the induction of labour. There are two key questions. Should all women be offered information on epidurals antenatally? Should an automatic check of women's knowledge of epidurals be made with all women on admission even if an epidural is not part of their birth plan lest participation in the decision to accept or refuse an unplanned epidural becomes necessary? Certainly, timely information provision seems crucial to enabling women to better understand the offer of an epidural and to empowering them to be active partners in the consent discourse shared decision‐making [38].

A strength of this study lies in the range of induction of labour methods and clinical indications, which facilitates more generalisable results. Data saturation was achieved, which is possible in qualitative studies with smaller sample sizes [39]. The generalisability of our results to other care facilities, however, remains open, although responses to this report may strengthen such generalisability.

We did not purposively sample for this small exploratory study cognisant that achieving meaningful representation across ethnic groups and socio‐economic status would best be achieved by conducting additional studies [40]. Though we did achieve some ethnic diversity in our sample, representative of patients attending the inner‐city hospital, non‐fluent English speakers were not included. The UK Confidential Enquiries into Maternal Deaths, however, suggest non‐English speakers are more likely to be poorly informed [41]. The inclusion criteria should be broadened in future investigations by use of a translator. The high level of educational attainment among participants requires consideration in the generalisability of the results and raises concern regarding how patients with lower health literacy experience the consent process. The issues that we have identified with English‐speaking women indicate that the situation is likely worse for non‐English‐speaking women and in this way our results contribute to addressing health inequalities.

Women on the postnatal and labour ward were interviewed; women who were induced and promptly discharged from the labour ward may have been eligible and may have had a contrasting experience. In practice, it is unlikely women did not attend the postnatal ward after having an epidural. As most women were interviewed within 4 days of delivery, there remains a risk of recall bias and we cannot be certain of the veracity of the accounts provided. The extended time from delivery to interview was due to difficulties in identifying a time that suited participants, particularly as they were primiparous women. To enable a fuller insight into the consent interactions, an ethnographic study examining anaesthetists' language choices throughout the consent process would inform recommendations to improve current practice.

This study suggests that the consent processes for epidural analgesia in the context of induction of labour are variable and often perceived to be of low quality by women. Failure of anaesthetists and midwives to adequately assess women's understanding of the risks, benefits and alternatives by anaesthetists and midwives was evidenced by women's poor understanding of these aspects of epidural analgesia in induction of labour. We believe our findings will not be unique to our institution, and we recommend an urgent review of the consent process for women in labour so that anaesthetists may be confident that patients in their care are providing truly informed consent.

## Supporting information


**Appendix S1.** Interview schedule.
