# Supplementary material for: Patient perception of consent processes for epidural analgesia in induction of labour: a qualitative study
Source: Anaesthesia. 2025 May 12;80(10):1199–206. doi: 10.1111/anae.16637 (PMC12434448; doi:10.1111/anae.16637)
Supplement: Supplementary file 1 — Appendix S1. Interview schedule. [file ANAE-80-1199-s001.docx]

**Appendix S1.** Interview schedule

**Purpose**

- What do you understand by consent?
- How does it relate to your view of you making decisions about your care?

**Initial Approach**

- How was the issue of consent raised with you?

**Who**

- How were you prepared when you were asked for your consent? (Were you given any preliminary information?)
- What do you feel your role was in the process?
- How was the purpose of the consent process explained to you?

**What – Content**

- In the context of consenting for epidural before inducing labour, what information was given to you regarding its purpose?
- Were you given any information sheets, websites or other sources of information?
- Can you recall your thoughts and plan for pain relief before being admitted for labour?
- If you had antenatal classes, where they private or public and what did you understand from them about pain relief in induced labour?
- Did your feelings towards epidural as a pain relief option change once induction conversations began?
- Were you told that induction of labour may be more painful than spontaneous labour, and would you say this influenced your decision to consent for epidural?
- What do you think are the important things to address when consenting a patient to epidural pain relief before inducing labour?
- Were risks discussed with you? How were they explained to you? Were you told how common/likely?
- Were benefits discussed with you? How were they explained to you?
- Did you feel consenting to an epidural was a choice?
- Did it cross your mind you could say no to epidural? Were the risks and benefits of this discussed?
- Can you recall discussion of alternative options to epidural?

**How – Process**

- What difficulties, if any, did you experience when you were asked for your consent?
- Did the doctor check your understanding when seeking consent? How?
- Did you get the opportunity to ask questions and if so, did you ask any?
- Did you feel satisfied by the answers you received?
- What do you think is the purpose of the consent form?
- In terms of the paperwork you were provided with:
- Were there any areas that you found confusing?
- Was it clear to you what you were giving your consent to?
- Was there any information that you would have liked to have known that you weren’t told before you made your decision?
- How well informed do you think you were about epidurals?
- Was there anything else you wanted to add about your experience?
- Would you liked anything to have been done differently in the consent process?
